# Recurrent unilateral anterior uveitis in an HLA-B27-positive ankylosing spondylitis patient: varicella-zoster virus etiology case report

**DOI:** 10.3389/fimmu.2026.1831499

**Published:** 2026-09-10

**Authors:** Xuan Liao, Junyi Lou, Ning Fan, Xuyang Liu

**Affiliations:** 1 Department of Ophthalmology, Affiliated Hospital of North Sichuan Medical College, Nanchong, Sichuan, China; 2 Medical School of Ophthalmology and Optometry, North Sichuan Medical College, Nanchong, Sichuan, China; 3 Shenzhen Eye Hospital, Shenzhen Eye Medical Center, Southern Medical University, Shenzhen, Guangdong, China; 4 Xiamen Eye Center, Xiamen Research Center for Eye Diseases and Key Laboratory of Ophthalmology, Xiamen University, Xiamen, Fujian, China; 5 Department of Ophthalmology, Shenzhen People’s Hospital (The First Affiliated Hospital, Southern University of Science and Technology), Shenzhen, Guangdong, China

**Keywords:** ankylosing spondylitis, aqueous humor assay, case report, HLA-B27, recurrent unilateral anterior uveitis

## Abstract

**Background:**

A 28-year-old Chinese woman with human leukocyte antigen (HLA)-B27-positive ankylosing spondylitis (AS) for 5 years experienced recurrent left-eye blurred vision and redness for 4 years (2–3 episodes/year). This was previously attributed to AS without etiologic testing. Her father and brother had AS but without ocular involvement. The patient received no systemic disease-modifying therapy for AS throughout her clinical course.

**Methods:**

Ophthalmic examinations [visual acuity, intraocular pressure (IOP), slit-lamp, and dilated fundus] and 100-µL left-eye aqueous humor testing [via paracentesis: varicella-zoster virus (VZV) immunoglobulin G (IgG), Goldmann–Witmer coefficient (GWC), and herpes simplex virus (HSV)-1/2/cytomegalovirus (CMV) antibodies/DNA] were performed. Targeted stepwise topical anti-inflammatory and IOP-lowering regimens were administered.

**Results:**

For the left eye, the following were observed: 20/30 visual acuity, 36-mmHg IOP, mutton fat keratic precipitates (KP), and iris atrophy. Findings for the right eye: were normal. Aqueous testing revealed elevated VZV IgG (1,227.02 U/mL) and a positive GWC (114.03), whereas HSV/CMV markers were negative. After treatment, visual acuity in the left eye improved to 20/20, IOP normalized, and symptoms resolved. No corneal sensation assessment was performed at any visit, and the patient denied any subjective corneal sensory discomfort during follow-up.

**Conclusion:**

Defaulting to AS-related uveitis in HLA-B27-positive patients should be avoided, and aqueous testing is recommended because it enables targeted management. Clinicians should adopt rational, stepped topical hypotensive combination therapy.

## 1 Introduction

Ankylosing spondylitis (AS) is a chronic inflammatory disorder strongly associated with HLA-B27, and acute anterior uveitis (AAU) is its most common extra-articular manifestation, affecting up to 33% (1) of patients with AS. In those with HLA-B27-positive recurrent unilateral anterior uveitis, the condition is typically attributed to the underlying rheumatic disease and often responds well to topical corticosteroids (2). However, viral etiologies—particularly varicella-zoster virus (VZV), herpes simplex virus, and cytomegalovirus—can produce a similar clinical picture but require distinct management strategies (3). Misdiagnosis of viral uveitis as AS-related AAU may lead to inappropriate immunosuppression and delayed antiviral therapy. Recent studies emphasize the value of aqueous humor analysis in differentiating infectious from non-infectious uveitis. Here, we describe a case of recurrent unilateral anterior uveitis in a patient with HLA-B27-positive AS who had no prior targeted ocular treatment before presenting to our hospital. Her condition was ultimately confirmed as VZV-associated uveitis by aqueous humor testing. This case highlights the importance of etiologic confirmation even in patients with a known autoimmune disease.

## 2 Case presentation

A 28-year-old Chinese woman presented to Shenzhen Eye Hospital with recurrent blurred vision and redness in her left eye in May 2020. The symptoms had persisted for 4 years in paroxysmal episodes, occurring 2 to 3 times annually, with complete recovery of vision during the intervening periods. Her medical history included back pain for 5 years of HLA-B27-positive AS, and previous evaluations attributed the uveitis to her underlying AS without definitive etiologic testing. Her father and brother also had AS but without ocular involvement. She denied any history of clinical varicella (chickenpox) infection or cutaneous herpes zoster (shingles) eruption, and had no history of systemic immunosuppressive therapy. No systemic medication targeting AS was prescribed in our hospital during the whole treatment period. Given the patient’s family history of AS and long-standing back pain, her ocular manifestations could be easily misdiagnosed as AS-associated iridocyclitis; however, the episodic pattern and complete normalization during intervening periods warranted further scrutiny.

**Figure 1:**
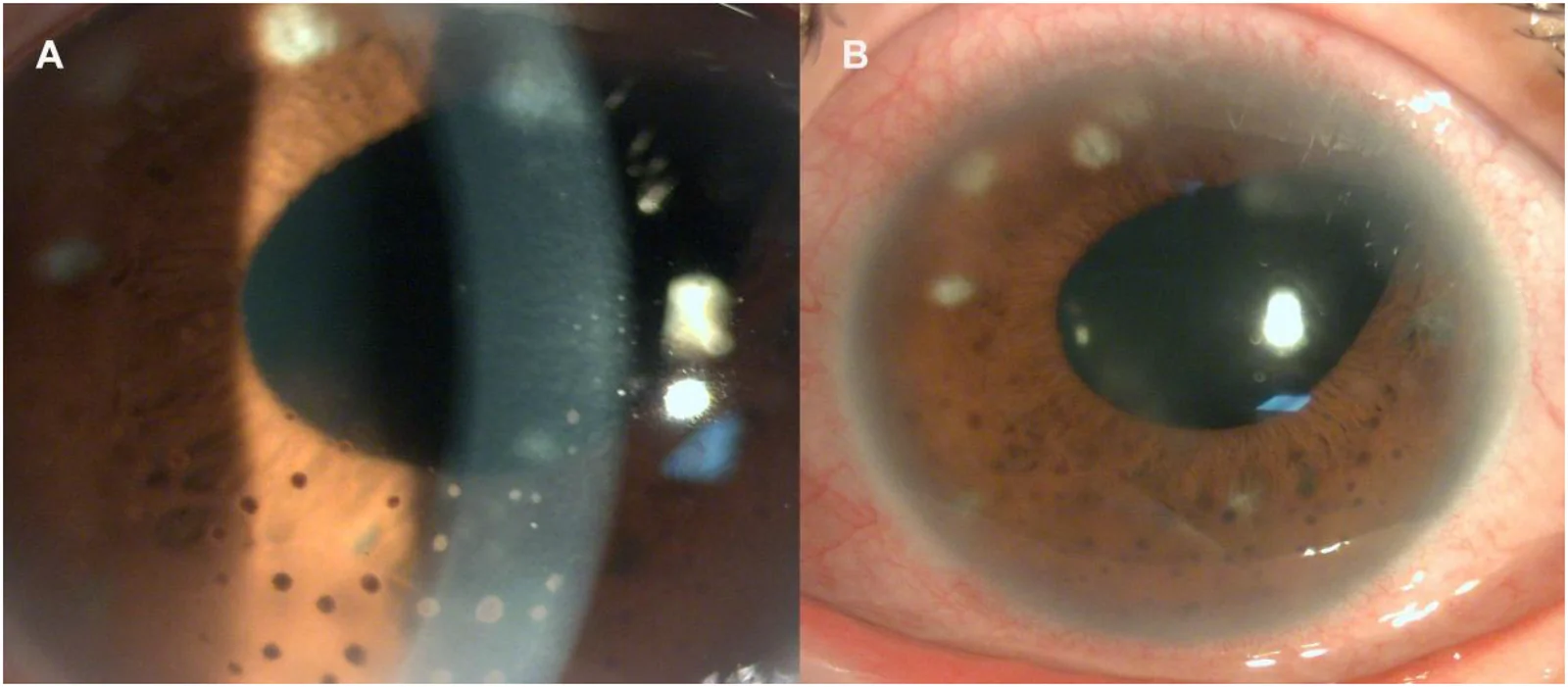
**(A)** Large, pigmented mutton fat keratic precipitates (KP) and **(B)** patchy iris atrophy with corectopia.

On ophthalmic examination, her best-corrected visual acuity was 20/20 in the right eye (OD) and 20/30 in the left eye (OS), with intraocular pressure (IOP) measured at 18 mmHg OD and 36 mmHg OS (normal range, 10–21 mmHg). Slit-lamp biomicroscopy revealed no abnormalities in the right eye. The left eye showed moderate circumcorneal injection, microcystic corneal edema, anterior chamber flare, and large, pigmented mutton fat keratic precipitates (KP) (**Figure 1A**) scattered over the inferior corneal endothelium. It also presented with patchy iris stromal atrophy in both nasal and temporal quadrants, corectopia with superior pupil displacement, and peripheral anterior synechiae (PAS) involving positions from 1 o’clock to 3 o’clock (**Figure 1B**). Dilated fundus examination was unremarkable bilaterally; optic discs and maculae were normal, and the peripheral retina showed no signs of vitritis or chorioretinal lesions. Corneal sensation testing was not performed during all clinical visits, and the patient never reported foreign body sensation, hypoesthesia, or other corneal sensory discomfort spontaneously.

These features appear inconsistent with the typical ocular findings of AS-associated iridocyclitis, which classically presents as non-granulomatous inflammation, small non-pigmented KP, and predominantly posterior synechiae, rather than iris atrophy or corectopia. Moreover, the patient’s clinical picture did not support a diagnosis of isolated iridocorneal endothelial (ICE) syndrome. Although the ICE syndrome may present with iris atrophy, corectopia, and PAS, it typically does not involve recurrent inflammatory attacks or anterior chamber flare. Given the patient’s history of AS, we considered several differential possibilities: ICE syndrome coexisting with AS, or ICE syndrome coexisting with glaucomatocyclitic crisis (Posner– Schlossman syndrome). However, none of these hypotheses fully accounted for the entire clinical presentation. To investigate the recurrent uveitis, a 100-μL sample of aqueous humor was obtained from the left eye via anterior chamber paracentesis on the day of her initial presentation in May 2020. Laboratory testing at the Eye Institute Clinical Laboratory showed a markedly elevated VZV IgG titer of 1,227.02 U/mL (normal reference value <16 U/mL) and a positive Goldmann–Witmer coefficient (GWC) of 114.03 (negative reference value ≤4), indicating local intraocular antibody production against VZV. In contrast, antibody titers for herpes simplex virus (HSV)-1, HSV-2, and cytomegalovirus (CMV) were undetectable, and reverse transcriptase–polymerase chain reaction for VZV, HSV-1, HSV-2, and CMV DNA returned negative results (<500 copies/mL), effectively ruling out active viral replication. These findings confirmed the diagnosis of VZV-associated anterior uveitis in the left eye with secondary ocular hypertension, excluding both AS-associated uveitis and other viral etiologies.

**Figure 2:**
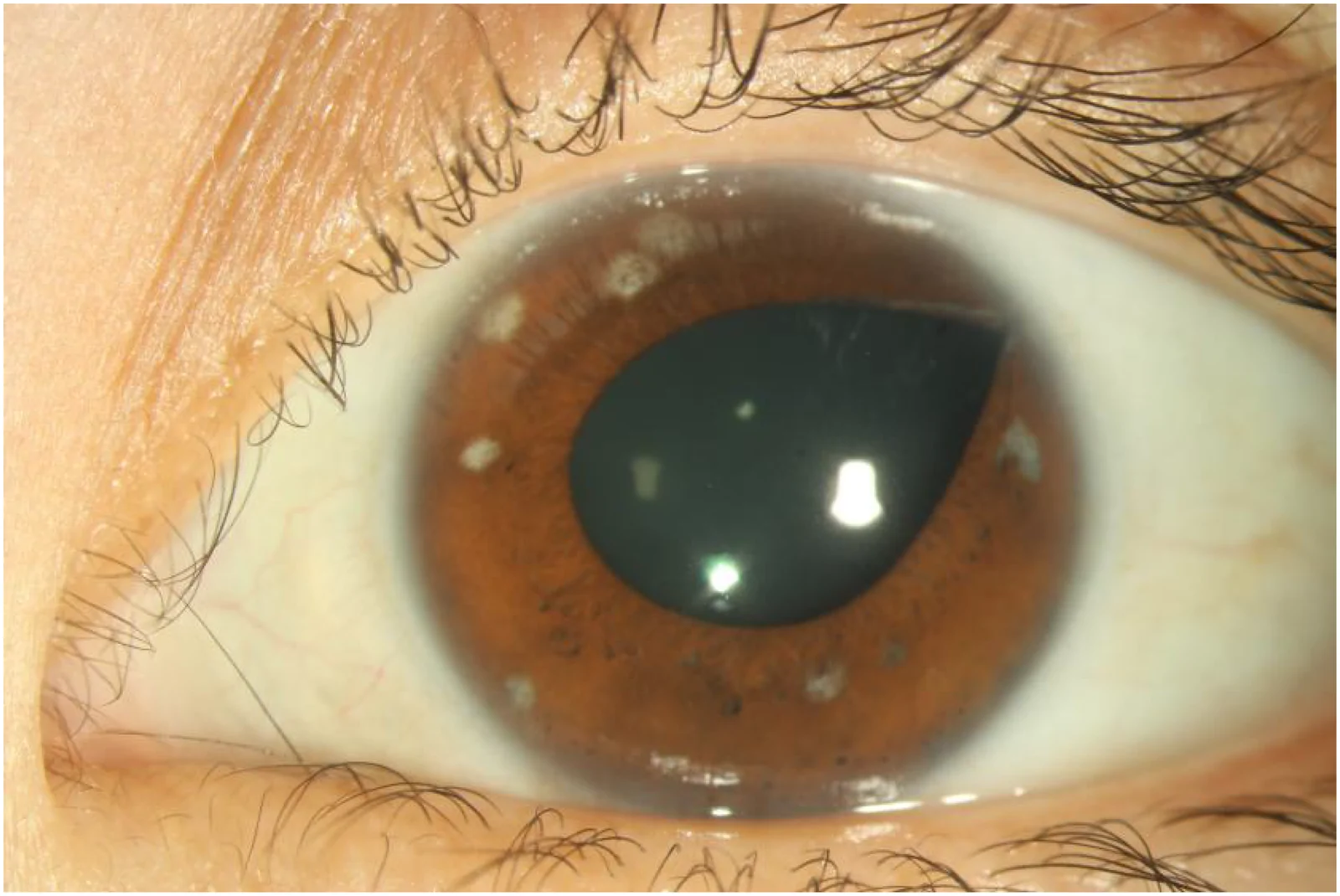
A decrease in the number of keratic precipitates (KP) during the intervening periods and still clear iris atrophy lesions.

The patient was prescribed topical prednisolone acetate 1% (Pred Forte) for anti-inflammatory treatment, combined with brimonidine tartrate (α_2_-adrenergic receptor agonist) 0.15% (Alphagan) plus brinzolamide (carbonic anhydrase inhibitor) 1%/timolol maleate (beta-adrenergic receptor blocker) 0.5% (Azarga) at standard clinical doses. This was combined with ganciclovir 0.1% eye drops (QID) and ganciclovir 0.15% ophthalmic gel (TID) for local antiviral prophylaxis against latent VZV reactivation. After 1 month of discharge maintenance therapy with Alphagan plus Azarga, the patient switched to self-administered carteolol hydrochloride 1% (Mikelan, BID) alone for convenience. Two weeks after the initiation of topical ocular anti-inflammatory and hypotensive medications, the patient reported resolution of her symptoms (**Table 1**). A repeat examination showed an improved best-corrected visual acuity of 20/20 in the left eye and normalized IOP at 18 mmHg. The slit-lamp examination revealed a clear cornea without edema, rare residual KP in the inferior endothelium, and no anterior chamber flare or cells, although the patchy iris atrophy, corectopia, and PAS persisted (**Figure 2**). Strict follow-up hospital visits were scheduled at 2 weeks, 1 month, and 2 months after the initial presentation, and close monitoring lasted only for 2 months upon completion of initial treatment. Subsequent long-term self-medication status was unclear due to a prolonged time interval and poor patient memory, and the patient suspended all topical eye medications spontaneously after May 2021 without further outpatient visits until the routine re-examination in May 2026.

**Table 1:** Clinical timeline.

Time	Chief complaint	Symptom manifestations	Outcome
2016 (24 years old)	Low back pain	AS	
2016–2020	Recurrent symptoms in the center eye	Decreased vision accompanied by red eyes, occurring 2–3 times a year, with complete recovery during the intervening periods	Misdiagnosed as AS-related uveitis; no systemic AS medication used during this period
May 2020	The first time to visit our hospital	Vision: right 20/20, center 20/30; IOP: right 18 mmHg, center 36 mmHg; KP, iris atrophy, pupil displacement, PAS; no corneal sensation examination performed	Suspected viral uveitis. An anterior chamber puncture was performed to confirm VZV-associated anterior uveitis
Two weeks after the treatment	First follow-up visit	Center eye vision is 20/20, IOP is 18 mmHg, and the inflammation has subsided	Atrophy of the iris and persistent presence of PAS

AS, ankylosing spondylitis; IOP, intraocular pressure; KP, keratic precipitates; PAS, peripheral anterior synechiae; and VZV, varicella-zoster virus..

Five years later, her best-corrected visual acuity was 1.0 (20/20) in both eyes; the cup-to-disc ratio was 0.3 bilaterally with normal optic disc morphology in both eyes. No signs of recurrent anterior uveitis were observed on slit-lamp biomicroscopy, and dilated fundus examination remained unremarkable. The patient reported no episodes of blurred vision, redness, or eye pain since 2021, and her underlying AS remained stable with no significant exacerbation of back pain.

## 3 Discussion

In most AS patients with typical presentations, defaulting to AS-associated uveitis is reasonable clinical practice; however, when atypical warning signs are present, clinicians should actively consider viral etiologies. The clinical findings documented in this case—large pigmented mutton-fat KP, patchy iris stromal atrophy, corectopia, PAS, and markedly elevated IOP (36 mmHg)—are not merely “atypical” for HLA-B27/AS-associated uveitis. In fact, these are characteristic features of viral anterior uveitis. Unilaterality, elevated IOP, decreased corneal sensation, and diffuse or sectoral iris atrophy are considered highly specific signs for HSV or VZV anterior uveitis by international expert consensus (4). Corneal swelling, iris atrophy, and raised IOP are explicitly listed as “clinical features suggestive of infection (herpes)” (5). One limitation of this study is the absence of corneal sensation measurement records throughout all visits; nevertheless, the patient never described subjective corneal hypoesthesia or abnormal ocular surface discomfort during clinical inquiry. Therefore, regardless of whether the patient has a known systemic autoimmune disease, the triad of granulomatous anterior uveitis, iris atrophy, and ocular hypertension should directly trigger a diagnostic workup for viral etiologies, including anterior chamber paracentesis with GWC/PCR testing.

The apparent discrepancy between negative VZV PCR and a markedly positive GWC (114.03) in this case requires careful interpretation. In recurrent or chronic VZV anterior uveitis, the virus typically exists in a state of latent reactivation rather than active replication. Consequently, the viral DNA load in aqueous humor may be very low, particularly when sampling occurs late in the disease course or during an intercritical period, potentially leading to false-negative PCR results. A recent cross-sectional study (6) of 81 eyes with unilateral anterior uveitis reported a PCR sensitivity of only 72.2% and a negative predictive value of 58.3% for detecting viral pathogens, indicating that a negative PCR result cannot reliably rule out viral etiology. In contrast, the GWC detects local intraocular antibody production, which persists even when viral replication is minimal or intermittent. In recurrent VZV uveitis, GWC has been shown to have higher diagnostic sensitivity than PCR, and a GWC > 4 is considered indicative of local intraocular antibody production (7). The remarkably elevated GWC of 114.03 in our patient provides unequivocal evidence of VZV involvement. The GWC formula inherently corrects for blood-aqueous barrier leakage by indexing specific antibody to total IgG in both aqueous humor and serum. Our patient’s GWC of 114.03 far exceeds the positive threshold, while simple barrier disruption typically yields only a mild elevation. The absence of aqueous antibodies against HSV/CMV despite their presence in serum further excludes non-specific leakage. Thus, a GWC of 114.03 unequivocally confirms intraocular VZV-specific antibody production. Therefore, the combination of a negative PCR and a positive GWC suggests a post-infectious or latent reactivation-related inflammatory process rather than acute active viral replication. This interpretation has important therapeutic implications: systemic antiviral therapy is not required, and treatment can focus on topical antiviral, anti-inflammatory, and IOP-lowering agents. This case also provides a practical medication reference: rational stepped therapy combining brinzolamide 1%/timolol maleate 0.5% (Azarga, a fixed carbonic anhydrase inhibitor/beta-adrenergic receptor blocker) with brimonidine tartrate 0.15% (Alphagan, an α_2_-adrenergic receptor agonist) serves as the preferred initial IOP-lowering regimen for severe secondary ocular hypertension. Following short-term inflammatory control, simplified maintenance monotherapy with carteolol hydrochloride 1% (Mikelan, a single-agent beta-blocker) may be implemented long-term to reduce systemic and ocular drug exposure burden. The patient’s successful management with the above BID-dosed topical regimen, followed by safe discontinuation in 2021 without disease recurrence, further validates this treatment strategy. The favorable clinical response to this regimen in our patient supports this approach. Notably, the nearly 5-year disease-free survival of uveitis and stable AS activity, confirmed by the comprehensive ophthalmic re-examination in May 2026 (including normal visual acuity and cup-to-disc ratio), demonstrates that etiologically targeted local therapy is sufficient to achieve long-term sustained remission in such cases, avoiding unnecessary systemic immunosuppression and its associated risks. No systemic anti-rheumatic or immunosuppressive treatment for AS was administered to this patient during the whole clinical management period, which avoided overlapping systemic immunosuppression.

The potential mechanisms underlying VZV reactivation in HLA-B27-positive AS patients can be explored from an immunogenetic perspective. As the strongest genetic risk factor for AS, HLA-B27 has been implicated in disease pathogenesis through three main hypotheses: molecular mimicry leading to the expansion of autoreactive CD8^+^ T cells, the formation of cell-surface homodimers that activate Th17 cells, and misfolding in the endoplasmic reticulum that triggers the unfolded protein response and upregulates IL-23 expression (8). The misfolding hypothesis is particularly relevant: endoplasmic reticulum (ER) stress-induced unfolded protein response (UPR) activation promotes the IL-23/IL-17 axis, creating a permissive inflammatory environment that may facilitate latent VZV reactivation. Furthermore, ERAP1/ERAP2 polymorphisms can influence HLA-B27-restricted antiviral CD8^+^ T-cell responses, potentially impairing immune surveillance against latent viruses. Collectively, these findings suggest that AS-associated immune dysregulation—including aberrant antigen presentation, ER stress, and IL-23/IL-17 axis overactivation—may disrupt immune surveillance of latent VZV infection, trigger viral reactivation, and ultimately lead to recurrent anterior uveitis.

This case highlights a critical clinical pitfall: defaulting to AS-related uveitis in HLA-B27-positive patients, risking overlooked viral etiologies, and inappropriate management strategies. AS is strongly associated with anterior uveitis, affecting approximately 20%–30% of AS patients. Notably, 91% of these cases are classified as non-infectious uveitis characterized by small, non-pigmented KP, with a prevalence of 121 cases per 100,000 (9). This is distinct from the granulomatous mutton fat KP and iris atrophy seen in VZV-associated anterior uveitis. Infectious uveitis is usually caused by HSV, VZV, and CMV (10).

The negative VZV RT-PCR in this case suggested a post-infectious or latent reactivation-related inflammatory process, eliminating the need for systemic antiviral therapy and focusing instead on local anti-inflammatory and hypotensive drugs. Importantly, this diagnosis avoided an unnecessary escalation of systemic AS therapy (e.g., tumor necrosis factor inhibitors), which would have been ineffective and exposed the patient to unnecessary immunosuppression. Clinicians should prioritize etiologic confirmation with aqueous humor testing in recurrent uveitis, even in patients with known AS, to ensure targeted management and optimize outcomes.

## Data Availability

The original contributions presented in the study are included in the article/supplementary material. Further inquiries can be directed to the corresponding author.
